# Femoral osteochondrosis mimicking chronic arthritis

**DOI:** 10.1186/1546-0096-12-S1-P288

**Published:** 2014-09-17

**Authors:** Blanca E Bica, Sergio Tapia, Aline Fraga

**Affiliations:** 1 Rheumatology, Universidade Federal do Rio de Janeiro, Rio de Janeiro, Brazil

## Introduction

Osteochondrosis or osteochondritis are alterations characterized by failure in sub-chondral ossification and affects the immature skeleton of children and teenagers. It may affects any epiphysis, apophysis or short bone with similar radiological and anatomopathological characteristics. It has no defined etiology, although there are some risk factors as constitutional predisposing, trauma and ischemia. Femoral osteochondrosis is usually unilateral and can be misdiagnosed as juvenile idiopathic arthritis (JIA). The authors report two patients with bilateral femoral condyle osteochondrosis that were treated as JIA.

## Objectives

To describe two cases of knee osteochondrosis in a same family that mimicked JIA.

## Methods

The first patient was a 10 years old boy who presented with right knee trauma history one year before and since then, he complained of pain and progressive swelling in both knees. Six months later, he presented significant local edema with flogistic signs and bilateral valgism deformity. He was submitted to tibia´s biopsy in other hospital showing no bone alterations at hystopathological examination. Laboratorial investigation revealed raise of acute phase inflammatory markers and normal total blood count, liver and kidney’s function and protein eletrophoresis. Research for falciform anaemia, auto-immune and mycobacterial diseases were negative. He was treated with NSAIDS, corticosteroids and MTX with no response. The computadorized tomography of knees showed bilateral distal femoral epyphyseal morphologic alterations characterized by height difference and lateral femoral condyle sclerosis with high irregularity at articular superficies, moderate joint effusion, bilateral supra-patellar bursa distention, synovial thickness with linear calcification. He was diagnosed with bilateral femoral condyle osteochondrosis. The patient was submitted to a surgical procedure to reduce articular deformity and epiphysiodesis of bilateral distal femur. After 5 years, when the patient was 15 years old, his cousin, who lived in a different city, showed similar symptoms of the same disease. In this case, the disease started at 8 years old, triggered by a trauma after falling in the ground. After one week, he started presenting progressive swelling and pain on the right knee. After one month, the left knee also presented significant local edema with flogistic signs and he was sent to a rheumatologic clinic. He was treated as JIA for one year with naproxen, MTX and prednisolone and developed severe bilateral valgism deformity. After knowing of the similarity of his cousin’s disease he came to our clinic and was diagnosed as bilateral femoral condyle osteochondrosis.

## Results

Osteochondrosis is a heterogeneous clinical condition regarding its clinical presentation and severity, and has been described in medical literature for a long time. The course of the history showed that the repetitive trauma and excessive stress may have a role in the pathogenesis of osteochondrosis (DOUGLAS, 1981). Another important etiologic factor to be considered is the insufficient blood supply. Several animal models studies have demonstrated an important association of inadequate perfusion of cartilaginous channels and the subsequent development of local osteochondrosis (YTREHUS, 2004).

## Conclusion

Although femoral bilateral osteochondrosis is very rare, general physicians, pediatricians and specially rheumatologists and orthopedic surgeons should be alert to this condition. Attention should be given to any child or teenager who complains of chronic articular pain or has growing disturbance because this condition may lead to severe deformities, as presented, and may affect considerably the quality of life.

## Disclosure of interest

None declared.

